# BLOOD-BASED CARDIAC BIOMARKERS AND PHYSICAL ACTIVITY: PRELIMINARY RESULTS

**DOI:** 10.1093/geroni/igae098.3475

**Published:** 2024-12-31

**Authors:** Jennifer Shearon, Baylie Rushing, Madeleine Love, Denise Head

**Affiliations:** Washington University in St. Louis, St. Louis, Missouri, United States; Washington University in St. Louis, St. Louis, Missouri, United States; Washington University in St. Louis, St. Louis, Missouri, United States; Washington University in St. Louis, St. Louis, Missouri, United States

## Abstract

Mixed findings regarding effects of physical activity on the brain in later adulthood motivate further research into mechanisms and moderators of potential effects. Cardiovascular health could be part of a mechanistic pathway and/or a moderator of physical activity effects. While there are myriad indicators of cardiovascular health, blood-based cardiac markers may prove particularly sensitive. One goal of this study was to investigate associations of two blood-based markers of cardiovascular health with physical activity/cardiorespiratory fitness. The second aim was to examine the moderating effect of cardiac biomarkers in associations of physical activity/fitness with neurotrophins, considered molecular mechanisms of physical activity effects, as well as with hippocampal volumes. Cognitively normal participants in the Knight ADRC Adult Children Study (n=56, ages 43-85) completed a blood draw for assessment of cardiac biomarkers (high sensitivity cardiac troponin T, N-terminal probrain natriuretic peptide) and neurotrophins (brain-derived neurotrophic factor, insulin-like growth factor, vascular endothelial growth factor). Composites were created for cardiac biomarkers and for neurotrophins. Participants completed a submaximal exercise test to estimate cardiorespiratory fitness and wore an actigraphy watch for seven days, from which a physical activity composite score was created. Hippocampal volumes were from the most recent MRI scan. Higher physical activity was associated with lower levels of cardiac biomarkers (p=0.03). There was no moderating effect of cardiac biomarkers on associations between physical activity and neurotrophin levels (p=0.74) or physical activity and hippocampal volumes (p=0.24). Blood-based cardiac biomarkers may be useful for considering mechanistic associations between physical activity and cardiovascular health in larger samples.

